# Human immunome, bioinformatic analyses using HLA supermotifs and the parasite genome, binding assays, studies of human T cell responses, and immunization of HLA-A*1101 transgenic mice including novel adjuvants provide a foundation for HLA-A03 restricted CD8^+^T cell epitope based, adjuvanted vaccine protective against *Toxoplasma gondii*

**DOI:** 10.1186/1745-7580-6-12

**Published:** 2010-12-03

**Authors:** Hua Cong, Ernest J Mui, William H Witola, John Sidney, Jeff Alexander, Alessandro Sette, Ajesh Maewal, Rima McLeod

**Affiliations:** 1 Department of Surgery(Ophthalmology and Visual Sciences), The University of Chicago, Chicago, Illinois 60637, USA; 2 Department of Parasitology, School of Medicine, Shandong University, Jinan, Shandong 250012, PR China; 3 Division of Vaccine Discovery, La Jolla Institute for Allergy and Immunology, La Jolla, California 92037, USA; 4 Pharmexa-Epimmune, San Diego, California 92121, USA; 5 Synthetic Biomolecules, San Diego, California 92121, USA; 6 Departments of Surgery (Ophthalmology and Visual Sciences) and Pediatrics (Infectious Disease), Committees on Immunology, Molecular Medicine, and Genetics, Institute of Genomics and Systems Biology, and The College, The University of Chicago, Chicago, Illinois 60637, USA

## Abstract

**Background:**

Toxoplasmosis causes loss of life, cognitive and motor function, and sight. A vaccine is greatly needed to prevent this disease. The purpose of this study was to use an immmunosense approach to develop a foundation for development of vaccines to protect humans with the HLA-A03 supertype. Three peptides had been identified with high binding scores for HLA-A03 supertypes using bioinformatic algorhythms, high measured binding affinity for HLA-A03 supertype molecules, and ability to elicit IFN-γ production by human HLA-A03 supertype peripheral blood CD8^+ ^T cells from seropositive but not seronegative persons.

**Results:**

Herein, when these peptides were administered with the universal CD4^+^T cell epitope PADRE (AKFVAAWTLKAAA) and formulated as lipopeptides, or administered with GLA-SE either alone, or with Pam_2_Cys added, we found we successfully created preparations that induced IFN-γ and reduced parasite burden in HLA-A*1101(an HLA-A03 supertype allele) transgenic mice. GLA-SE is a novel emulsified synthetic TLR4 ligand that is known to facilitate development of T Helper 1 cell (TH1) responses. Then, so our peptides would include those expressed in tachyzoites, bradyzoites and sporozoites from both Type I and II parasites, we used our approaches which had identified the initial peptides. We identified additional peptides using bioinformatics, binding affinity assays, and study of responses of HLA-A03 human cells. Lastly, we found that immunization of HLA-A*1101 transgenic mice with all the pooled peptides administered with PADRE, GLA-SE, and Pam_2_Cys is an effective way to elicit IFN-γ producing CD8^+ ^splenic T cells and protection. Immunizations included the following peptides together: KSFKDILPK (SAG1_224-232_); AMLTAFFLR (GRA6_164-172_); RSFKDLLKK (GRA7_134-142_); STFWPCLLR (SAG2C_13-21_); SSAYVFSVK(_SPA250-258_); and AVVSLLRLLK(SPA_89-98_). This immunization elicited robust protection, measured as reduced parasite burden using a luciferase transfected parasite, luciferin, this novel, HLA transgenic mouse model, and imaging with a Xenogen camera.

**Conclusions:**

*Toxoplasma gondii *peptides elicit HLA-A03 restricted, IFN-γ producing, CD8^+ ^T cells in humans and mice. These peptides administered with adjuvants reduce parasite burden in HLA-A*1101 transgenic mice. This work provides a foundation for immunosense based vaccines. It also defines novel adjuvants for newly identified peptides for vaccines to prevent toxoplasmosis in those with HLA-A03 supertype alleles.

## Background

Toxoplasmosis is a disease of major medical importance. *Toxoplasma gondii *causes congenital infections responsible for stillbirths and spontaneous abortions[1-5]. In addition, it causes neurologic disorders, uveitis, and systemic infections in immune-compromised patients. *Toxoplasmic *encephalitis is a cause of morbidity and mortality in those with congenital disease and persons with AIDS[6]. *T. gondii *is acquired by consumption of lightly cooked meat, especially lamb and pork contaminated with bradyzoites[7,8] or by ingestion of food or water contaminated with oocysts containing sporozoites, which are the product of a sexual cycle in the intestine of the cat [8,9].

Development of an effective vaccine would prevent this disease. Attenuated *T. gondii *tachyzoites (e.g., S48, TS-4, T-203, ΔRPS13)have been employed for live vaccinations of non-human animals[10-15]. Parasites attenuated by knockout of gene transcription recently have been proposed as a new type of attenuated vaccine candidate[13-15]. However, safety considerations may limit vaccination of humans with live organisms. Development of peptide-based vaccines created with epitopes which elicit IFN-γ production by CD8^+ ^T cells[16] is a promising strategy to mobilize the immune system against *T. gondii *in humans[16-20]. Thus, an effort to create an immunosense epitope-based vaccine was initiated.

Recently, three peptide epitopes, KSFKDILPK (SAG1_224-232_), AMLTAFFLR (GRA6_164-172_), and RSFKDLLKK (GRA7_134-142_), were found by our group to elicit IFN-γ from peripheral blood mononuclear leukocytes (PBMCs) from *T. gondii *seropositive HLA-A03 supertype humans but not from PBMCs of *T. gondii *seronegative HLA-A03 supertype humans[18]. Herein, initially we designed 4 CD8^+ ^T cell epitope containing vaccine formulations comprised of lipopeptides that incorporate PADRE as well as a novel oil-in-water emulsion that includes a specially formulated synthetic MLA derivative called GLA-SE[21-24]. First, efficacy of various vaccine formulations consisting of the three previously identified CD8^+ ^T cell peptides eliciting HLA-A*1101-restricted, CD8^+^T cell-mediated IFN-γ production *in vitro *from HLA-A*1101 mice was examined.

Then, in order to identify additional peptides from *T. gondii *tachyzoites, bradyzoites and sporozoites of Type I and Type II strains that are effective in eliciting IFN-γ from HLA-A03 supertype restricted CD8^+ ^T cells, the first step was to select proteins with biologic properties, such as being secreted, compatible with MHC Class I processing. Then, bioinformatic algorithms to identify HLA-A03 supertype bound peptides were utilized to find additional, novel *T. gondii*-derived, potential CD8^+ ^T cell eliciting epitopes restricted by the HLA-A03 supertype. We screened peptides from tachyzoite, bradyzoite and sporozoite proteins (GRA10, GRA15, SAG2C, SAG2D, SAG2X, SAG3, SRS9, BSR4, SPA, MIC) of the type II *T. gondii *strain, ME49, searching for those with high binding scores in a bioinformatic analysis (IC_50 _< 50 nM) and then in binding assays. In addition, peripheral blood mononuclear cells from seropositive and seronegative persons were tested for response to these peptides using an IFN-γ ELISpot assay. These latter studies were performed to attempt to identify other peptides that would be promising candidates for inclusion in a multi-epitope, next generation immunosense vaccine. Then, information obtained from testing the first three peptides studied was used to guide formulation and administration of a peptide pool. This pool included the first three peptides identified earlier and tested in the initial experiments combined with the newly identified peptides that elicited IFN-γ from human HLA-A03 supertype restricted CD8^+ ^T cells. All the peptides in this pool were tested with a universal T helper epitope called PADRE and a new, promising adjuvant called GLA-SE with Pam_2_Cys in HLA-A*1101 transgenic mice. Capacity to induce IFN-γ production by spleen CD8^+ ^T cells, and to protect against parasite burden following subsequent challenge were determined. For parasite challenge, a luciferase transfected Type II Prugneaud parasite was administered, followed by luciferin administration and imaging with a Xenogen camera system a week later. This allows detection and quantization of bioluminescent parasites as a biomarker to assess efficacy of immunizations in protection.

## Results

### Construction of CD4-CD8 lipopeptide based candidate vaccine

**Figure 1:**
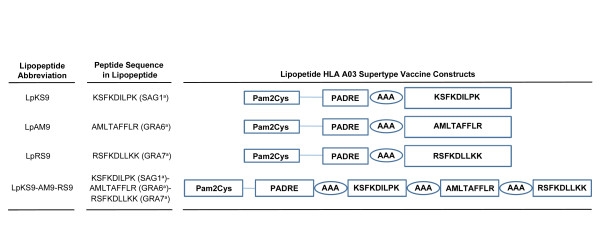
**Schematic representation of the synthetic lipopeptide immunogens used in this study**. The C-terminal end of a promiscuous CD4^+ ^T cell peptide epitope (PADRE) was joined in sequence with the N-terminal end of one of three different *T. gondii *CD8 T cell epitopes: SAG1_224-232 _(A), GRA6_164-172 _(B); GRA7_134-142 _(C) or three epitopes linked together (D) with a three alanine linker. The N-terminal end of each resulting CD4-CD8 peptide was extended by a lysine covalently linked to one molecule of palmitic acid. This results in a four lipopeptides construct. The abbreviation Lp is used throughout the manuscript whenever the lipopeptide (Lp) has been studied. When there is a mixture of components or undivided components they are named individually. Sequence of PADRE is AKFVAAWTLKAAA. Structure of Pam_2_Cys is PAM_2_KSS. a = abbreviation for name of protein from which peptide is derived.

Three CD8^+ ^T cell epitopes had been identified from *T. gondii *proteins, based on their significant recognition by T cells from *T. gondii *seropositive HLA-A03 individuals[18]. A universal CD4^+ ^T cell epitope, PADRE (AKFVAAWTLKAAA), was linked in sequence with the N-terminal end of each of the three different *T. gondii *CD8^+ ^T cell epitopes: KSFKDILPK (SAG1_224-232_), AMLTAFFLR (GRA6_164-172_), RSFKDLLKK (GRA7_134-142_). Also, these three epitopes were linked together with three alanines as the linker. The N-terminal end of each resulting CD4-CD8 peptide or polypeptide was extended by a lysine covalently linked to two molecules of the palmitic acid moiety. The lipopeptides (Lp) were named as LpKS9, LpAM9, LpRS9 and LpKS9-AM9-RS9. They are shown in Figure 1.

### Immunogenicity of lipopeptides in HLA-A*1101 transgenic mice

**Figure 2:**
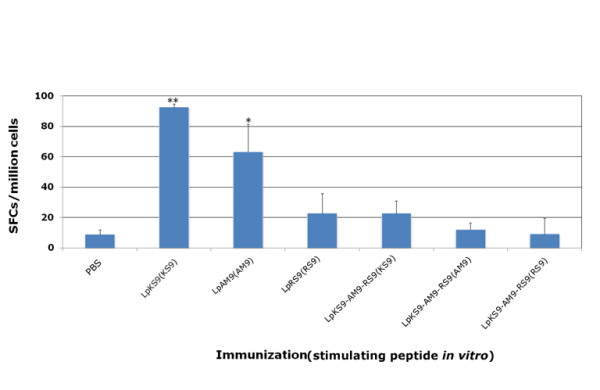
HLA-A*1101 mice immunized with lipopeptides. Mice were immunized with lipopeptides: LpKS9, LpAM9, LpRS9 and LpKS9-AM9-RS9 which were shown in Figure 1. The mice were immunized twice at intervals of three weeks. Ten to fourteen days after the last immunization, spleen cells were separated from immunized mice and stimulated by appropriate peptides in an *ex vivo *IFN-γ ELISpot assay. Data presented are averages of three independent replicate experiments. *, *P *< 0.05; **, *P *< 0.01.

HLA-A*1101 transgenic mice were immunized twice at intervals of three weeks with lipopeptides which were administered in PBS. Two weeks after the last immunization, the spleens were removed from immunized mice and the ability of splenocytes to produce IFN-γ upon stimulation with peptides was analyzed. Transgenic mice immunized with the three single peptide lipopeptide vaccines had T cells that produced IFN-γ (Figure 2). The lipopeptide vaccines LpKS9 and LpAM9 stimulated higher IFN-γ production than LpRS9 (Figure 2). However, LpKS9-AM9-RS9 with three peptide epitopes linked together did not stimulate strong IFN-γ responses when splenocytes from these mice were exposed to each of the individual peptides *in vitro*. Only results with LpKS9(KS9) and LpAM9(AM9) achieved statistical significance (Figure 2).

### Comparison of vaccination with different formulations of peptides

**Figure 3:**
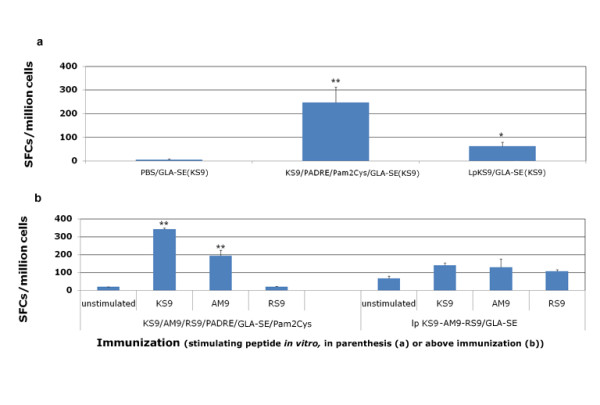
**Lipopeptides vaccine compared with peptide pool vaccine**. HLA-A*1101 transgenic mice were immunized either with lipopeptides or a mixture of peptides plus GLA-SE and Pam_2_Cys. LpKS9 and KS9/PADRE/GLA-SE/Pam_2_Cys (Figure 3a), LpKS9-AM9-RS9 and KS9/AM9/RS9/PADRE/GLA-SE/Pam_2_Cys (Figure 3b) were used as immunogens. For the lipopeptide immunizations, the mice were vaccinated twice at intervals of three weeks. For the immunizations containing individual components including the same peptides, the mice were vaccinated three times at intervals of two weeks. Ten to fourteen days after the last immunization, spleen cells were separated from immunized mice and stimulated by the appropriate peptide in an *ex vivo *IFN-γ ELISpot assay. Data presented are a representative example from three independent experiments. *, *P *< 0.05; **, *P *< 0.01.

In order to determine which formulation was most immunogenic, vaccination with a single peptide or a mixture of the peptides was compared with linked lipopeptide vaccines. Results of a representative experiment are presented in Figure 3. Mice immunized with a vaccine formulated with a single peptide SAG1_224-232_, KS9-PADRE with the adjuvancy of GLA-SE and Pam_2_Cys, elicited IFN-γ production (SFC:248 ± 65; mean ± SEM) four-fold higher (p < 0.01) than mice that received LpKS9 GLA-SE vaccination (63 ± 17)(Figure 3a). Similar results, with substantial IFN-γ production, were found when splenocytes from mice immunized with a mixture of peptides and adjuvants. These results were compared with IFN-γ production by splenocytes from mice immunized with a lipopeptide vaccine constructed with the three peptides linked together with alanine spacers LpKS9-AM9-RS9. Significant IFN-γ production was only present with KS9 and AM9 stimulation of spleen cells, and was much less robust and not significant when the spleen cells were stimulated by RS9 peptide.

### Effects of adjuvant on immunogenicity of pooled peptide vaccination

**Figure 4:**
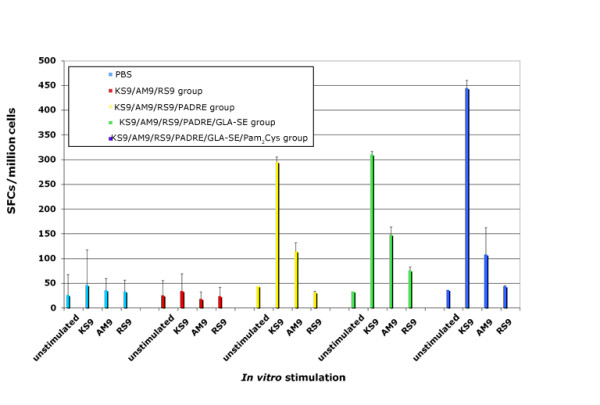
HLA-A*1101 transgenic mice immunized with peptide pool and adjuvants. Mice were immunized with PBS, peptide pool, peptide pool with PADRE, peptide pool with PADRE in GLA-SE, peptide pool with PADRE and Pam_2_Cys in GLA-SE. Splenic T cells were isolated 10-14 days post-immunization and exposed to each peptide in an *ex vivo *IFN-γ ELISpot assay.

To determine whether, and if so how, adjuvants effected immunogenicity of these peptides, HLA-A*1101 transgenic mice were immunized with pools of peptides that included all of the three peptides (KS9, AM9, RS9) alone or with varying adjuvants. HLA-A*1101 transgenic mice were immunized with: (1) CD8^+ ^epitope peptide pool, (2) peptide pool plus PADRE, (3) peptide pool plus PADRE emulsified with GLA-SE, and (4) peptide pool plus PADRE and Pam_2_Cys emulsified with GLA-SE. Mice were inoculated three times at intervals of two weeks. Eleven to fourteen days post immunization, spleen cells were isolated and exposed to each individual peptide. A peptide was considered immunogenic if it induced IFN-γ spot formation that was significantly higher in the immunization group compared with the group inoculated with PBS. After the immunizations, only KS9 and AM9 were found to be immunogenic in HLA-A*1101 transgenic mice, and only when the iniversal CD4^+ ^helper T cell peptide epitope PADRE was included. Robust responses were observed when GLA-SE was added in the vaccine. Greater responses were elicited when Pam_2_Cys was used as an adjuvant for some peptides but did not enhance responses to all of them, and in fact reduced the effect of some peptides. Figure 4 shows the representative data of IFN-γ spot formation from the four immunization groups which were stimulated by individual peptides.

### Vaccination with peptide pools and adjuvants protects mice against type II parasite challenge

**Figure 5:**
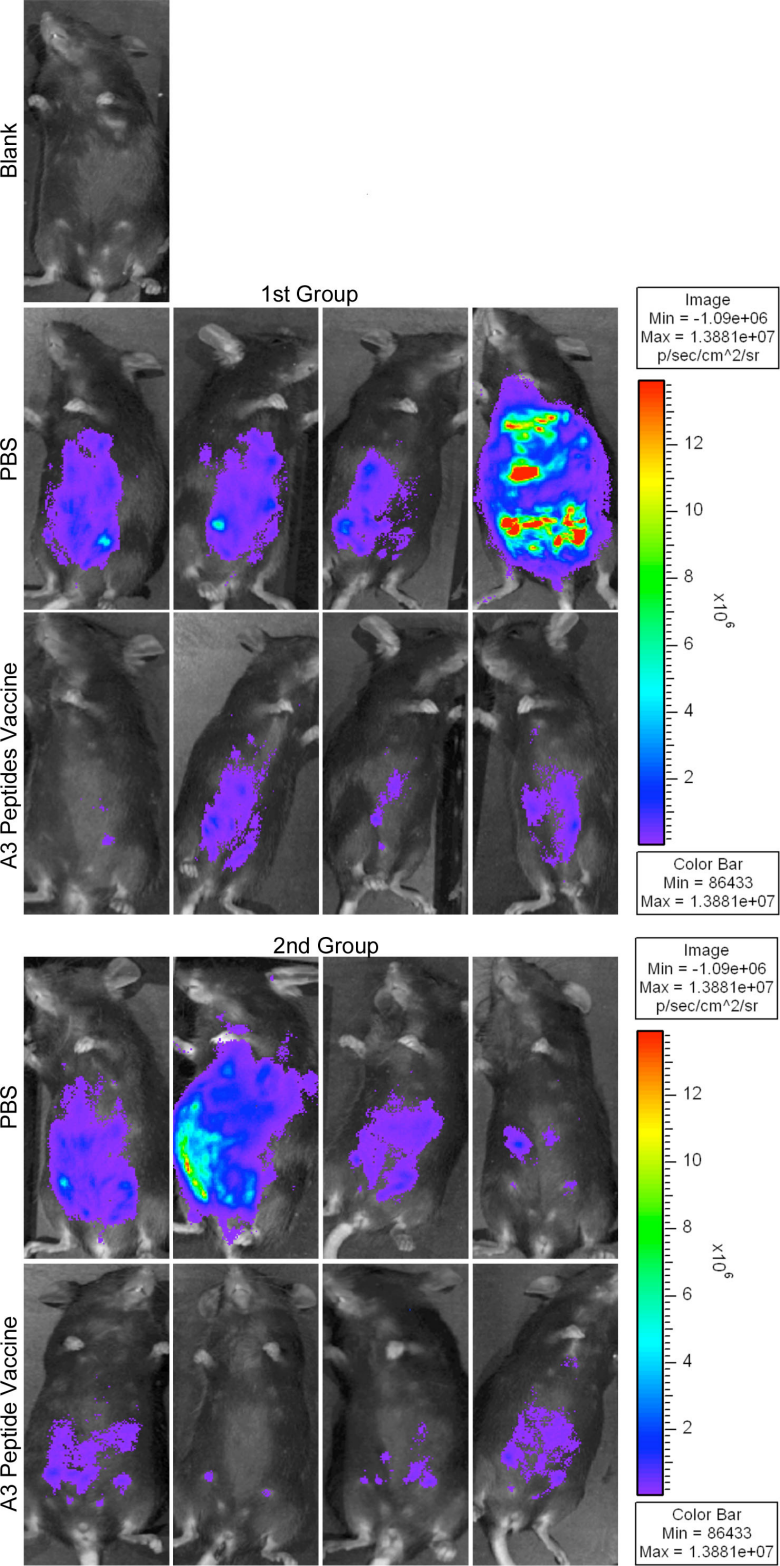
***In vivo *protection demonstrated with imaging using Xenogen camera**. HLA-A*1101 transgenic mice immunized with peptide pool and adjuvants were protected compared to control mice inoculated with PBS when they were challenged with 10,000 Prugneaud strain (Fluc)-*T. gondii *luciferase expressing parasites. There were a total of 5-9(usually 4-5 per group) mice tested in each control or immunization group. Differences between control and immunization groups were significant(p < 0.0064 using natural log transformed data and two-sample t test).

HLA-A*1101 transgenic mice were immunized with peptide pools plus PADRE and Pam_2_Cys in GLA-SE three times at intervals of two weeks. Mice were challenged 2 weeks after the last immunization. They were imaged 7 days after they had been challenged with 10,000 Pru (Fluc) using the Xenogen *in vivo *imaging system. As shown in Figure 5, numbers of luciferase expressing parasites in immunized HLA-A*1101 transgenic mice were significantly less compared to numbers of parasites in unimmunized mice. Mean [standard deviation](median) was 136[253](30) million for nonimmunized mice versus 5[4](5) million for immunized mice. With natural log transformed data, means[standard deviations] were 3.6[1,6] for nonimmunized mice versus 1.4[.8] for immunized mice. Differences were significant (e.g., p < 0.0064, for the pooled experiments, using natural log transformed data and two-sample t test).

### Identification of new candidate *T. gondii *specific HLA-A*1101-restricted epitopes

**Figure 6:**
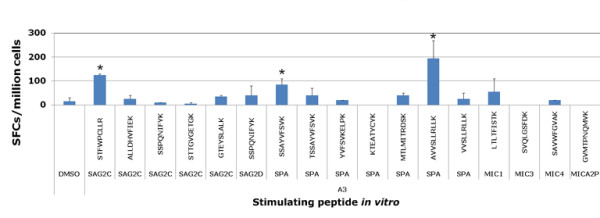
**New peptides tested with PBMCs from HLA-A03 seropositive and seronegative donors**. Then Peripheral blood mononuclear cells from seropositive *T. gondii *donors were tested for response to these predicted HLA-A03 supertype restricted CD8^+ ^T cell epitope individual peptides by using IFN-γ ELISpot assay. Peptides that induced significant IFN-γ spot formation compared to DMSO are denoted by an asterisk. *, *P *< 0.05.

We initially identified 3 epitopes that provided protection against parasite challenge. As a next generation vaccine that might be even more robust, elicit CD8^+^T cells that would be effective against all three life cycle stages and Type I and II genetic types of the parasite, we sought to identify additional HLA-A11 epitopes to be used for vaccine development. In order to identify additional peptides from *T. gondii *that were present in tachyzoites, bradyzoites, and sporozoites of Type II strains for HLA-A03 supertype restricted CD8^+ ^T cells, we screened candidate peptides from tachyzoite, bradyzoite and sporozoite proteins (GRA15, GRA10, SAG2C, SAG2X, SAG3, SRS9, SPA and MIC) of the Type II *T. gondii *strain (Tables 1 and 2). For parsimony in numbers of peptides utilized, we attempted to include peptides present in Type I as well as Type II parasite genetic types. Peripheral blood mononuclear cells from seropositive *T. gondii *donors were tested for response to these peptides by using the IFN-γ ELISpot assay. Pooled peptides were tested initially. Pools 2 and 5 were significantly different (p < 0.05) from the control (data not shown). Then the individual peptides were tested (Figure 6). Three out of 34 epitopes elicited responses greater than 50 IFN-γ SFC from seropositive donors PBMC, but not from seronegative donors PBMC (Figure 6). These peptides and the proteins from which they are derived are: STFWPCLLR (SAG2C_13-21_); SSAYVFSVK (SPA_250-258_); and AVVSLLRLLK (SPA_89-98_) adding proteins expressed in sporozoites and bradyzoites to the peptides selected. All peptides identified herein then were found to show high binding affinity to three to five HLA-A03 supertype alleles in the MHC-peptide binding assay (Table 3). The numbers in Table 3 which indicate high binding affinity are those less than 500 IC_50 _nM. They are indicated by bolded font numbers in Table 3.

**Table 1:** Peptides, their affinity for HLA-A*1101, and their immunogenicity in humans and for transgenic mice

Peptide Sequences	Protein^1^	Affinity^2 ^HLA-A*1101	Elicit IFN-γ^3 ^in		Immunogenicity^4 ^in mice
				
			Seropositive human	Seronegative human	
					
KSFKDILPK	SAG1_224-232_	54	+	-	+
AMLTAFFLR	GRA6_164-172_	3.6	+	-	+
RSFKDLLKK	GRA7_134-142_	14	+	-	-
STFWPCLLR	SAG2C_13-21_	10	+	-	+
SSAYVFSVK	SPA_250-258_	10	+	-	+
AVVSLLRLLK	SPA_89-98_	34	+	-	+

^1^Peptides derived from these proteins and the position within the proteins

^2^Binding affinity was performed by MHC binding assay.

^3^PBMC from four *T. gondii*-seropositive HLA-A03 supertype persons and four seronegative persons were stimulated with peptides, the T cell that produce IFN-γ were tested by ELISpot assay.

^4^Splenic T cell were isolated from HLA-A*03 supertype(which includes the HLA-A*1101 haplotype) mice 10 to 14 days after peptide immunization and tested for their ability to generate IFN-γ in response to peptide.

**Table 2:** Predicted peptide candidates utilized for screening CD8+ T cells1

HLA-A*1101	ANTIGEN	PEPTIDE SEQUENCES	LENGTH	LOCATION	PREDICTED IC_50 _nM	POOL
HLA-A*1101	GRA15	STSPFATRK	9	152-160	5	P1
HLA-A*1101	GRA15	ASTSPFATRK	10	150-159	18.2	
HLA-A*1101	GRA10	AAAATPGLPK	10	568-577	9	
HLA-A*1101	GRA10	AAATPGLPK	9	569-577	13.2	
HLA-A*1101	GRA10	GVPAVPGLLK	10	507-516	17	
HLA-A*1101	GRA10	SVVEENTMAK	10	865-874	25.1	
HLA-A*1101	SAG2C	STFWPCLLR	9	13-21	9.3	P2
HLA-A*1101	SAG2C	ALLDHVFIEK	10	231-240	11.9	
HLA-A*1101	SAG2C	SSPQNIFYK	9	290-298	12.9	
HLA-A*1101	SAG2C	STTGVGETGK	10	163-172	28.4	
HLA-A*1101	SAG2C	GTEYSLALK	9	136-144	35.4	
HLA-A*1101	SAG2D	SSPQNIFYK	9	122-130	12.9	
HLA-A*1101	SAG2D	ALLEHVFIEK	10	63-72	20.2	P3
HLA-A*1101	SAG2D	SSAQTFFYK	9	290-298	1.8	
HLA-A*1101	SAG2D	TVYFSCDPK	9	154-162	5.3	
HLA-A*1101	SAG2D	PSSAQTFFYK	10	289-298	17.9	
HLA-A*1101	SAG3	VVGHVTLNK	9	80-88	15.4	
HLA-A*1101	SAG3	KQYWYKIEK	9	145-153	19.3	
HLA-A*1101	SRS9	TTCSVLVTVK	10	357-366	20.6	P4
HLA-A*1101	SRS9	AAASVQVPLK	10	140-149	29.9	
HLA-A*1101	SRS9	AIQSQKWTLK	10	169-178	35	
HLA-A*1101	BSR4	TTRFVEIFPK	10	284-293	10.3	
HLA-A*1101	BSR4	VSGSLTLSK	9	83-91	21.1	
HLA-A*1101	SPA	SSAYVFSVK	9	250-258	9.3	P5
HLA-A*1101	SPA	TSSAYVFSVK	10	249-258	12.2	
HLA-A*1101	SPA	YVFSVKELPK	10	253-262	18	
HLA-A*1101	SPA	KTEATYCYK	9	262-270	18.4	
HLA-A*1101	SPA	MTLMITRDSK	10	195-204	25.5	
HLA-A*1101	SPA	AVVSLLRLLK	10	89-98	6.5	
HLA-A*1101	SPA	VVSLLRLLK	9	90-98	7.2	
HLA-A*1101	MIC1	LTLTFISTK	9	338-346	13.8	P6
HLA-A*1101	MIC3	SVQLGSFDK	9	32-40	12.9	
HLA-A*1101	MIC4	SAVWFGVAK	9	16-24	17.2	
HLA-A*1101	MICA2P	GVMTPNQMVK	10	63-72	14.3	

^1 ^Peptides derived from GRA10, GRA15, SAG2C, SAG2D, SAG2X, SAG3, SRS9, BSR4, SPA, MIC1, MICA2P were screened for potential supertype epitopes using the ARB algorithms from immunoepitope database at http://www.iedb.org/ on the basis of their predicted binding affinity to HLA-A*1101. A total of 34 unique peptides IC_50 _< 50 nM (lower score, higher predicted binding affinity) of all ranked nonameric or decameric peptides were selected.

**Table 3:** MHC binding affinity assay for peptides that were identified with predictive algorithms for HLA-A03 supertype

				HLA-A03-SUPERTYPE						
					
PEPTIDE SEQUENCE	LENGTH	PROTEIN	POSITION	HLA-A*0301	HLA-A*1101	HLA-A*3001	HLA-A*3101	HLA-A*3301	HLA-A*6801	SUPERTYPE ALLELES BOUND
STFWPCLLR	9	SAG2C	13-21	**22**	**10**	1272	**1.2**	**3.4**	**0.97**	5
SSAYVFSVK	9	SPA	250-258	**12**	**10**	1826	**97**	**181**	**2.1**	5
AVVSLLRLLK	10	SPA	89-98	**17**	**34**	**8.1**	1296	1100	**94**	4

### Vaccination with peptide pools including newly identified peptides and adjuvants elicits IFN-γ and provides more protection to mice against Type II parasite challenge

**Figure 7:**
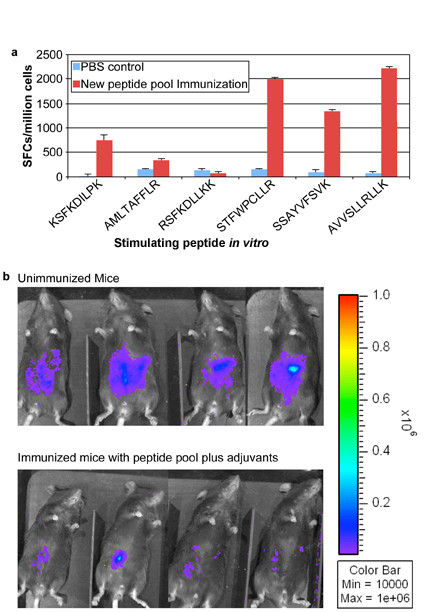
Addition of peptides to pool robustly protects HLA-A*1101 mice against Type II parasite challenge. HLA-A*1101 transgenic mice were immunized with PBS (controls) or peptide pool with PADRE and Pam_2_Cys in GLA-SE. Splenic T cells were isolated 10-14 days post immunization and exposed to each peptide in an *ex vivo *IFN-γ ELISpot assay (Figure 7a). HLA-A*1101 transgenic mice immunized with peptide pool and adjuvants were protected compared with control mice inoculated with PBS when they were challenged with 10,000 Pru (Fluc)-*T. gondii *luciferase expressing parasites (Figure 7b). Mice were immunized and in a subgroup immune function was studied at the same time as the challenge shown was performed. Differences between control and immunized mice were significant (p < 0.0064 using natural log transformed data and two-sample t test).

Then, HLA-A*1101 transgenic mice were immunized with peptide pools which included these newly identified peptides: KSFKDILPK (SAG1_224-232_); AMLTAFFLR (GRA6_164-172_); RSFKDLLKK (GRA7_134-142_); STFWPCLLR (SAG2C_13-21_); SSAYVFSVK (SPA_250-258_); AVVSLLRLLK (SPA_89-98_) plus PADRE and Pam_2_Cys in GLA-SE, They were immunized three times at intervals of two weeks. Significant IFN-γ spot formation responses were observed *in vitro *by the cells from immunized mice exposed to all the peptides except GRA7_134-142_. Figure 7a shows the representative data of IFN-γ spot formation from the four immunization groups which were stimulated by individual peptides. The inclusion of the newly identified peptides thus had potential to enhance immunogenicity in transgenic HLA-A*1101 mice.

Mice were challenged 2 weeks after the last immunization. They were imaged five days after they had been challenged with 10,000 Pru (Fluc) using a Xenogen *in vivo *imaging system. As shown in the initial experiment in Figure 7b, the numbers of luciferase expressing parasites in immunized HLA-A*1101 mice were significantly reduced compared to the numbers of parasites in unimmunized mice. Results were mean[standard deviation](median) 2.5[1](2.3) million for nonimmunized mice versus.6[.3](.5)million for immunized mice. With natural log transformed data, mean[standard deviation} was.9 [.4] for nonimmunized mice versus -.6[.4] for immunized mice; p < 0.0025 using natural log transformed data and two-sample t test.

### Population Coverage prediction

An algorithm was developed to calculate projected population coverage of a T cell epitope-based vaccine using MHC binding or T cell restriction data and HLA gene frequencies[17]. We used this web-based tool http://www.iedb.org/ to predict population coverage of these HLA-A03 supertype peptide epitopes-based vaccine. The population coverage calculation results in Table 4 indicate that such coverage is varied in different geographic regions. The HLA-A03 supertype molecules that present these peptides would be expected to be present in 28.90% population in Australia; 41.46% in Europe; 11.29% in North America; 18.86% in North-East Asia; 37.73% in South-East Asia; 34.29% in South-West Asia; 34.09% in Oceania; 27.32% in North Africa; 13.14% in Sub-Saharan Africa and 22.13% for others. The average population coverage by the HLA-A03 supertype is 24.51% ± 12.06%.

**Table 4:** Prediction of population coverage

	Class I		
	
Population/Area	Coverage ^a^	Average hit ^b^	PC90 ^c^
Australia	28.90%	1.30	0.14
Europe	41.46%	0.98	0.17
North Africa	27.32%	0.45	0.14
North America	11.29%	0.24	0.11
North-East Asia	18.86%	0.74	0.12
Oceania	34.09%	1.69	0.15
Other	22.13%	0.59	0.13
South America	0.42%	0.00	0.10
South-East Asia	37.73%	1.84	0.16
South-West Asia	34.29%	1.09	0.15
Sub-Saharan Africa	13.14%	0.23	0.12
**Average (Standard deviation)**	**24.51% (12.06%)**	**0.83 (0.58)**	**0.14 (0.02)**

**^a ^**projected population coverage for SAG1224-232 (KSFKDILPK); GRA6164-172 (AMLTAFFLR);GRA7134-142 (RSFKDLLKK); SAG2C13-21 (STFWPCLLR); SPA250-258(SSAYVFSVK); SPA89-98 (AVVSLLRLLK).

**^b ^**average number of epitope hits/HLA combinations recognized by the population

**^c ^**minimum number of epitope hits/HLA combinations recognized by 90% of the population.

Population coverage was calculated using the analysis resource available at the IEDB http://www.iedb.org, and as described by Bui et al. 2006 [PMID 16545123]. Calculations are based on HLA genotypic frequencies obtained from the dbMHC database http://www.ncbi.nlm.nih.gov/mhc/. Population coverage reflects the fraction of individuals predicted to respond the epitope set inclusive of SAG1224-232 (KSFKDILPK), GRA6164-172 (AMLTAFFLR), GRA7134-142 (RSFKDLLKK), SAG2C13-21 (STFWPCLLR), SPA250-258 (SSAYVFSVK), and SPA89-98 (AVVSLLRLLK). The value shown corresponds to the average coverage across 11 different major geographical areas.

## Discussion

In this study, we first evaluated immunization of HLA-A*1101 transgenic mice with either mixtures of peptides or lipopeptides derived from three identified *T. gondii *specific HLA-A*1101 restricted CD8^+ ^T cell epitopes emulsified in 3-deacylated monophosphoryl lipid A(GLA-SE) adjuvant. Immunizations of transgenic mice with a mixture of CD8^+ ^epitope peptide pools plus PADRE and adjuvants were able to induce splenocyte to produce IFN-γ and to protect against challenge with high numbers of Type II parasites.

Conjugation of CD8^+ ^T cell determinants to lipid groups is known to enhance specific cell-mediated responses to target antigens in experimental animals and humans[25-29], although mechanisms whereby immunity is achieved remains poorly understood. Lipopeptides hold several advantages over other conventional vaccine formulations; for instance, they are self-adjuvanting and display none of the toxicity-associated side effects of other Th1-inducing adjuvant systems. In our work, transgenic mice that were immunized with three short lipopeptide vaccines had T cells that produced IFN-γ. Among them the lipopeptide vaccine formulated with KS9 or AM9 stimulated higher IFN-γ production than the lipopeptide vaccine formulated with RS9. Unexplained and variable responses have been observed to high affinity binding peptides in other models, e.g., studies of Livingstone, Alexander, Sette et al with Lassa fever virus. It will be of interest to better understand possible mechanisms for such lack of response in future studies. However, the lipopeptides with three epitope peptides linked together with alanine spacers did not stimulate an IFN-γ response by splenocytes from immunized mice when the splenocytes subsequently were exposed to each of the peptides *in vitro*. The reason why the lipopeptides with the three linked peptides did not work well in the transgenic mice might be related to a frame shift caused by the linkers that altered the response to the original peptides rather than the alanines functioning for the intended purpose of introducing a cleavage motif. The three linker "AAA'' between the peptides had previously been demonstrated in other systems to result in sensitization to each linked peptide. However, surprisingly, it did not appear to work well herein.

Because the three linked peptides in the lipopeptide formulation were not effective and we had found that a mixture of the components with a single peptide was as or more robust than the lipopeptide, we tried this approach with the three peptides that had been included in the linked lipopeptide with the universal helper CD4^+ ^T cell peptide, PADRE, and adjuvants as described below. The response was robust both *in vitro *and *in vivo *(Figures 4 and 5).

Some studies have shown palmitoylated lipopeptide constructs to elicit long-lived, protective cellular responses against a variety of pathogens, including Hepatitis B virus (HBV), influenza virus, and *Plasmodium falciparum *[25-29]. Our work herein shows that mice immunized with mixture of CD8^+ ^and CD4^+ ^eliciting peptides and lipid Pam_2_Cys emulsified in GLA-SE elicited higher IFN-γ production than mice immunized with lipopeptides constructed with the same components of CD4^+ ^and CD8^+ ^eliciting peptides, and Pam_2_Cys. The approach using cocktails of non-covalently linked lipid mixed to helper T lymphocytes(HTL) and CD8^+^T cell (cytolytic T lymphcyte[CTL] and IFN-γ eliciting) epitopes for simultaneous induction of multiple CD8^+^T specificities would have significant advantages in terms of ease of vaccine development.

HTL responses are crucial for the development of CD8^+^T responses, at least in the case of lipidated covalently or non-covalently linked HTL-CTL epitope constructs formulated in PBS. Several previous studies have illustrated a role for CD4^+ ^responses for development of CD8^+^CTL responses, both in humans and in experimental animals[30-35]. The inclusion of PADRE, a synthetic peptide that binds promiscuously to variants of the human MHC class II molecule DR and is effective in mice, also augmented CD8^+ ^T cell effector functions by inducing CD4^+ ^T helper cells[30-35]. Both CD4^+ ^and CD8^+ ^epitopes were targeted in order to drive a protective immune response[34,35].

Adjuvanting antigens contributes to the success of vaccination. An example herein is that 3-deacylated monophosphoryl lipid A(GLA-SE), a detoxified derivative of the lipopolysaccharide (LPS) from *Salmonella minnesota R595 *was a potent adjuvant. This GLA-SE is a novel adjuvant which was formulated in an emulsion[21-24]. This is a Toll-like receptor 4 (TLR4) agonist that is a potent activator of Th1 responses [21-24]. It has been used as an adjuvant in human vaccine trials for several infectious disease and malignancy indications. It has been very effective as an adjuvant providing CD4^+ ^T cell help for immunizations against other protozoan infections such as leishmaniasis [21-24]. In our study, a robust response was observed when GLA-SE was included in preparation for immunization of mice. Pam_2_Cys (S-[2,3-bis(palmitoyloxy)propyl]cysteine) is a lipid component of macrophage-activating lipopeptide. Pam_2_Cys binds to and activates dendritic cells by engagement of Toll-like receptor 2 (TLR-2)[24]. Toll-like receptors (TLRs) function as pattern-recognition receptors in mammals[36]. We have found that both TLR2 and TLR4 receptors participate in human host defense against *T. gondii *infection through their activation by GPIs and GIPLs(Melo, Hargrave, Miller, Blackwell, Gazinelli, McLeod et al, in preparation, 2010). TLR2 and TLR4 likely work together with other MyD88-dependent receptors, including other TLRs, to elicit an effective host response against *T. gondii *infection[36]. In our study, there was a slightly more robust response observed when Pam_2_Cys was co-administered for some peptides, but not all of them.

The goal of the present study was to identify HLA-restricted epitopes from *T. gondii *and evaluate whether they could provide protection against parasite challenge measured as protection against a luciferase producing Type ll parasite using a Xenogen camera system. In the future, additional more detailed studies involving analyses over longer times, other strains of the parasite and challenge with life cycle stages, evaluation of multiple organs including eye and brain, studies of protection in congenital infections, comparisons of delivery of these peptides as DNA encoding them versus other formulations. This future work will follow up and extend these intitial studies of reduction of parasite burden seen in Figures 5 and 7.

Various peptide-based approaches to induction of IFN-γ responses were evaluated as part of ongoing efforts to develop immunosense vaccines for use in humans with each of the supermotifs which would in total include more that 99% of the human population worldwide. Robust protection was achieved in the HLA-A*1101 transgenic mice challenged with Type II parasites following immunizations. In order to identify additional peptides from *T. gondii *that were present in tachyzoites or bradyzoites[37,38] or sporozoites of Type I and II strains and elicited IFN-γ from HLA-A03^+ ^supertype (which includes the HLA*1011 allele) restricted CD8^+ ^T cells, bioinformatic algorithms were utilized to identify novel, *T. gondii*-derived, epitopes restricted by the HLA-A03 supertype. Then PBMC cells were tested to determine whether the peptides elicited IFN-γ from human CD8^+ ^T cells from seropositive persons. This was intended to collectively provide broad coverage for the human population with HLA-A03 supertype worldwide. The additional peptides we identified as immunogenic for human peripheral blood cells were also robust in eliciting IFN-γ from splenocytes of HLA-A*1101 mice and protection when used to immunize these mice. These findings will facilitate development of an immunosense epitope-based vaccine for human use.

## Conclusion

A human immunome-based and parasite genome based bioinformatices approach was used to define candidate HLA-A03 supertype restricted peptides. Immunogenicity of a group of *T. gondii *HLA-A03 supertype restricted peptides, and therefore the proteins from which they are derived, for immune humans and HLA-A*1101 transgenic mice was demonstrated. These peptides elicit interferon-γ production by human CD8^+^T cells. They also elicit interferon γ production by mouse splenocytes when utilized to immunize HLA-A*1101 transgenic mice with a lipopeptide with a universal CD4^+ ^T cell eliciting epitope, PADRE, or in peptide pools with PADRE, Pam_2_Cys and GLA-SE, a novel adjuvant. Immunization studies demonstrate the need for and the efficacy of adjuvants in immunization of these HLA transgenic mice. Immunogenic peptides included KSFKDILPK (SAG1_224-232_); AMLTAFFLR (GRA6_164-172_); and RSFKDLLKK (GRA7_134-142_); STFWBCLLR (SAG2C_13-21_); SSAYVFSVK (SPA_250-258_); and AVVSLLRLLK (SPA_89-98_). The studies herein provide a foundation for immunosense based vaccines to prevent toxoplasmosis in those with the HLA-A03 supertype and information about how they can be adjuvanted.

## Methods

### Peptides and lipopeptides

HLA-A03 supertype CD8^+ ^T cell epitopes included: KSFKDILPK (SAG1_224-232_), AMLTAFFLR (GRA6_164-172_), and RSFKDLLKK (GRA7_134-142_). PADRE (AKFVAAWTLKAAA) was the universal CD4 helper peptide used in vaccine constructs. Pam_2_Cys (Pam_2_-KSS) also was included. Lipopeptide constructs used in this study are shown in Figure 1. Peptides and lipopeptides were synthesized by Synthetic Biomolecules, San Diego at > 90% purity. Additional HLA-A03 supertype bound peptides and their initial grouping into pools for *in vitro *studies are shown in Tables 1 and 2. A TLR4 agonist, a GLA-SE adjuvant, was synthesized by the Infectious Diseases Research Institute (Seattle, Washington) as a stable oil-in-water emulsion. AMLTAFFLR (GRA6 _164-172_) and additional new peptides were first dissolved in DMSO and then diluted in PBS.

### Mice

HLA-A*1101/K^b ^transgenic mice were produced at Pharmexa-Epimmune (San Diego, CA) and bred at the University of Chicago. These HLA-A*1101/K^b ^transgenic mice express a chimeric gene consisting of the 1 and 2 domains of HLA-A*1101 and the 3 domain of H-2K^b^, and were created on a C57BL/6 background. For each test, we used 4-5 mice for each group. Each experiment was repeated 2 to 3 times. Experiments in Figure 7b were performed including a subgroup analyzed for immune response in parallel with a subgroup in the challenge shown. All studies were conducted with approval of the Institutional Animal Care and Use Committee at the University of Chicago.

### Parasites

Transgenic *T. gondii *used for *in vivo *challenges was derived from Type II Prugniaud (Pru) strain and expresses the firefly luciferase (FLUC) gene constitutively by tachyzoites and bradyzoites. It was created, and kindly provided by S. Kim, J. Boothroyd and J. Saeij(Stanford University) and was maintained and utilized as previously described[18,37,39].

### Immunizations and challenge

To evaluate peptide immunogenicity, HLA-A*1101 transgenic mice were inoculated subcutaneously (s.c.) at the base of the tail using a 30-gauge needle with single peptides or a mixture of CD8^+ ^T cell peptides (50 μg of each peptide per mouse) and PADRE (AKFVAAWTLKAAA) emulsified in 20 μg of GLA-SE (TLR4 agonist) with or without Pam_2_Cys. Pam_2_Cys concentration was 5 mg/ml. For immunization with lipopeptides, HLA-A*1101 mice received 20 nmol lipopeptide dissolved in PBS or emulsified in GLA-SE. As controls, mice were injected with PBS or PBS/GLA-SE. For the lipopeptide immunizations, the mice were vaccinated twice at intervals of three weeks. For the peptide immunizations, mice were immunized three times at intervals of two weeks. For challenge studies, mice were immunized with peptide emulsions and challenged intraperitoneally (i.p.) 14 days post-immunization using 10,000 Type II parasites.

### *In vivo *bioluminescence imaging

Mice infected with 10,000 Pru-FLUC tachyzoites were imaged 7 days post-challenge using the *in vivo *imaging system (IVIS; Xenogen, Alameda, CA). Mice were injected i.p. with 200 μl of D-luciferin, anesthetized in an O_2_-rich induction chamber with 2% isoflurane, and imaged after 12 minutes. Photonic emissions were assessed using Living image^® ^2.20.1 software (Xenogen). Data are presented as pseudocolor representations of light intensity and mean photons/region of interest (ROI). All mouse experiments were repeated at least twice. There were 4-5 mice for each group. In the experiment in Figure 7b a subgroup of mice was used for studying immune response in parallel with the subgroup in the challenge shown.

### ELISpot assay

#### Murine splenocytes

Mice were euthanized 7 to 14 days after immunization. Spleens were harvested, pressed through a 70 μm screen to form a single-cell suspension, and depleted of erythrocytes with AKC lysis buffer (160 mM NH_4_Cl, 10 mM KHCO_3_, 100 M EDTA). Splenocytes were washed twice with Hank's Balanced Salt Solution (HBSS) and resuspended in complete RPMI medium (RPMI-1640 supplemented with 2 mM L-GlutaMax [Invitrogen], 100 U/ml penicillin, 100 μg/ml streptomycin, 1 mM sodium pyruvate, 50 M -mercaptoethanol, and 10% FCS) before they were used in subsequent *in vitro *assays. Peptide concentration was 20 mg/ml and 0.20 μl was used per well. ELISpot assays with murine splenocytes were performed using α-mouse IFN-γ mAb (AN18) and biotinylated α-mouse IFN-γ mAb (R4-6A2) as the cytokine-specific capture antibodies. Antibodies were monoclonal antibodies. 5 × 10^5 ^splenocytes were plated per well.

#### Human PBMC

PBMC were obtained, HLA haplotype was determined, and they were processed and cryopreserved as described[18]. ELISpot assays with human PBMCs were similar to those with murine splenocytes but used α-human IFN-γ mAb (1-D1K) with biotinylated α-human IFN-γ mAb (7B6-1) with 2 × 10^5 ^PBMCs per well. All antibodies and reagents used for ELISpot assays were from Mabtech (Cincinnati, OH). Antibodies were monoclonal antibodies. Both murine and human cells were plated in at least 3 replicate wells for each condition. Results were expressed as number of spot forming cells (SFCs) per 10^6 ^PBMCs or per 10^6 ^murine splenocytes.

### Bioinformatic predictions and MHC-peptide binding assays

Protein sequences derived from GRA10, GRA15, SAG2C, SAG2D, SAG2X, SAG3, SRS9, BSR4, SPA, and MIC were analyzed for CD8^+ ^T cell epitopes based on predicted binding affinity to HLA-A03 supertype molecules using ARB algorithms from immunoepitope database (IEDB) http://www.immuneepitope.org[40,41]. A total of 34 unique peptides IC_50 _< 50 nM of all ranked nonameric peptides were selected. All protein sequences were from ToxoDB 5.1.

Quantitative assays to measure binding of peptides to HLA class I molecules are based on inhibition of binding of radiolabeled standard peptide. Assays were as described[42]. Concentration of peptide yielding 50% inhibition of binding of radiolabeled probe peptide (IC_50_) was calculated. Under conditions used, where [radiolabeled probe] < [MHC] and IC_50 _≥ [MHC], measured IC_50 _values are reasonable approximations of true *K*_d _values[43,44].

### Statistical analyses

Statistical analyses for all *in vitro *assays were performed using 2-tailed student's T test. Natural log transformed data and two-sample t test were used to analyze data shown in Figures 5 and 7b. Two-tailed *P *values < 0.05 were considered statistically significant. Peptides were considered immunogenic in mice if they induced IFN-γ spot formation from immunized mice that were significant (*P *< 0.05) compared with spot formation from control mice. All mouse experiments were repeated at least twice. There were 4-5 mice for each group. The experiment in Figure 7b determined immune response and imaged mice in parallel.

## Competing interests

The authors declare that they have no competing interests.

## Authors' contributions

All authors contributed to the work in this manuscript. HC, EJM, WHW, JS, JA, AS, AM, and RM designed and/or performed experiments, provided key reagents, revised the manuscript for significant context, and reviewed the manuscript as submitted. HC, EJM, JS, JA, AM, and RM wrote the entire manuscript and edited drafts of the manuscript for significant content. All authors read and approved the final version submitted.
